# Effects of High-Temperature Growth of Dislocation Filter Layers in GaAs-on-Si

**DOI:** 10.1186/s11671-022-03762-9

**Published:** 2022-12-19

**Authors:** HoSung Kim, Dae-Myeong Geum, Young-Ho Ko, Won-Seok Han

**Affiliations:** 1 Optical Communication Components Research Section, Photonic/Wireless Devices Research Division, Electronics and Telecommunications Research Institute, Daejeon, Korea; 2 Quantum Optics Research Section, Quantum Technology Research Department, Electronics and Telecommunications Research Institute, Daejeon, Korea

**Keywords:** GaAs-on-Si, Dislocation filter layers, MOCVD, InAs quantum dots, Leakage currents

## Abstract

GaAs-on-Si templates with two different dislocation filter layers (DFLs) were grown at 550 °C low-temperature (LT)-DFL and 660 °C high-temperature (HT)-DFL using metal organic vapor-phase epitaxy and the effects of the growth temperature were studied. The threading dislocation density (TDD) values of LT-DFL and HT-DFL were 5.2 × 10^7^ cm^−2^ and 1.5 × 10^7^ cm^−2^, respectively. The 1.5 × 10^7^ cm^−2^ of TDD in HT-DFL is reduced by almost one order compared to the 1.2 × 10^8^ cm^−2^ of that in the control sample without DFLs. The annihilation process was mainly observed in the HT-DFL by a transmission electron microscope, resulting in a lower TDD. The 500-nm-thick GaAs bulk layer and InAs QDs were regrown on GaAs-on-Si templates and the optical properties were also evaluated by photoluminescence (PL). The highest PL peak intensity of the HT-DFL indicates that less non-radiative recombination in both the GaAs bulk and QDs occurred due to the reduced TDD. The GaAs *p*–*i*–*n* diodes were also fabricated to analyze the bulk leakage (*J*_B_) and the surface leakage current. The *J*_B_ of HT-DFL shows the lowest value of 3.625 × 10^–7^ A/cm^−2^ at applied bias voltage of 1 V, which is 20 times lower than the *J*_B_ of the control sample without DFLs. This supports that the high-temperature growth of DFL can make a good performance GaAs device on Si.

## Introduction

On the basis of benefits including novel functionalities, cost reduction and miniaturization, III-V-on-Si laser diodes (LDs) have been vigorously studied in the field of silicon photonics in both industrial and academic settings. The direct growth method [1] and the use of wafer bonding [2] are the two main technologies to make III-V-on-Si LDs. Direct growth accompanies many issues such as dislocation generation caused by a lattice mismatch. Nonetheless, compared to wafer bonding, it offers the significant advantage of allowing large-scale integration. The direct growth approach is consequently considered an ultimate goal for integration [3].

Recently, significant progress in the epitaxial direct growth of an InAs/GaAs quantum dot laser diode (QDLD) on a Si substrate by molecular beam epitaxy (MBE) has been achieved [4–11]. One of the key techniques to prepare a QDLD on a Si substrate is growing a high-quality GaAs buffer layer on Si substrate. To reduce threading dislocation density (TDD) caused by lattice mismatch between the GaAs and Si, numerous methods has been explored. Among them, the dislocation filter layers (DFLs) have been shown to reduce the TDD remarkably [12–16]. While many different materials for the DFL has been used to bend or block the direction of threading dislocation (TD), strained DFLs, which are composed of strained layers and superlattice layers such as InGaAs/GaAs, have been the most effective reducing the TDD [17–19]. This is because the misfit dislocation (MD) caused by the strained DFLs can annihilate the TD by combining two TDs into one MD.

Considering the movement of dislocations, a TD with high glide velocity may interact actively with a MD. To produce a TD with high glide velocity and a high likelihood to interact with a MD at the interface of DFLs, strained DFLs should be grown at high temperature. In a MBE system, the InGaAs layer is typically grown at around 530 °C, which is lower than the temperature of GaAs layer growth (580 °C). However, metal organic vapor-phase epitaxy (MOVPE) can grow both low-temperature (LT) and high-temperature (HT) InGaAs. In addition, MOVPE is considered the standard epitaxy system for large-scale production in industry. Hence, the growth of a QDLD on a Si substrate by MOVPE is advantageous in the silicon photonic industry.

In this paper, we grew GaAs-on-Si templates with two different DFLs, grown at 550 °C and 660 °C and studied the effects of the growth temperature. The TDD level was measured by electron channeling contrast imaging (ECCI) and a transmission electron microscope (TEM) was observed to understand the dislocation interaction. The optical properties of a regrown 500 nm GaAs layer and InAs quantum dots (QDs) grown by MOVPE on a GaAs-on-Si template were also evaluated by photoluminescence (PL). In addition, the electrical properties of regrown GaAs *p*–*i*–*n* diodes were analyzed by dark current measurement.

## Methods/Experimental

**Fig. 1:**
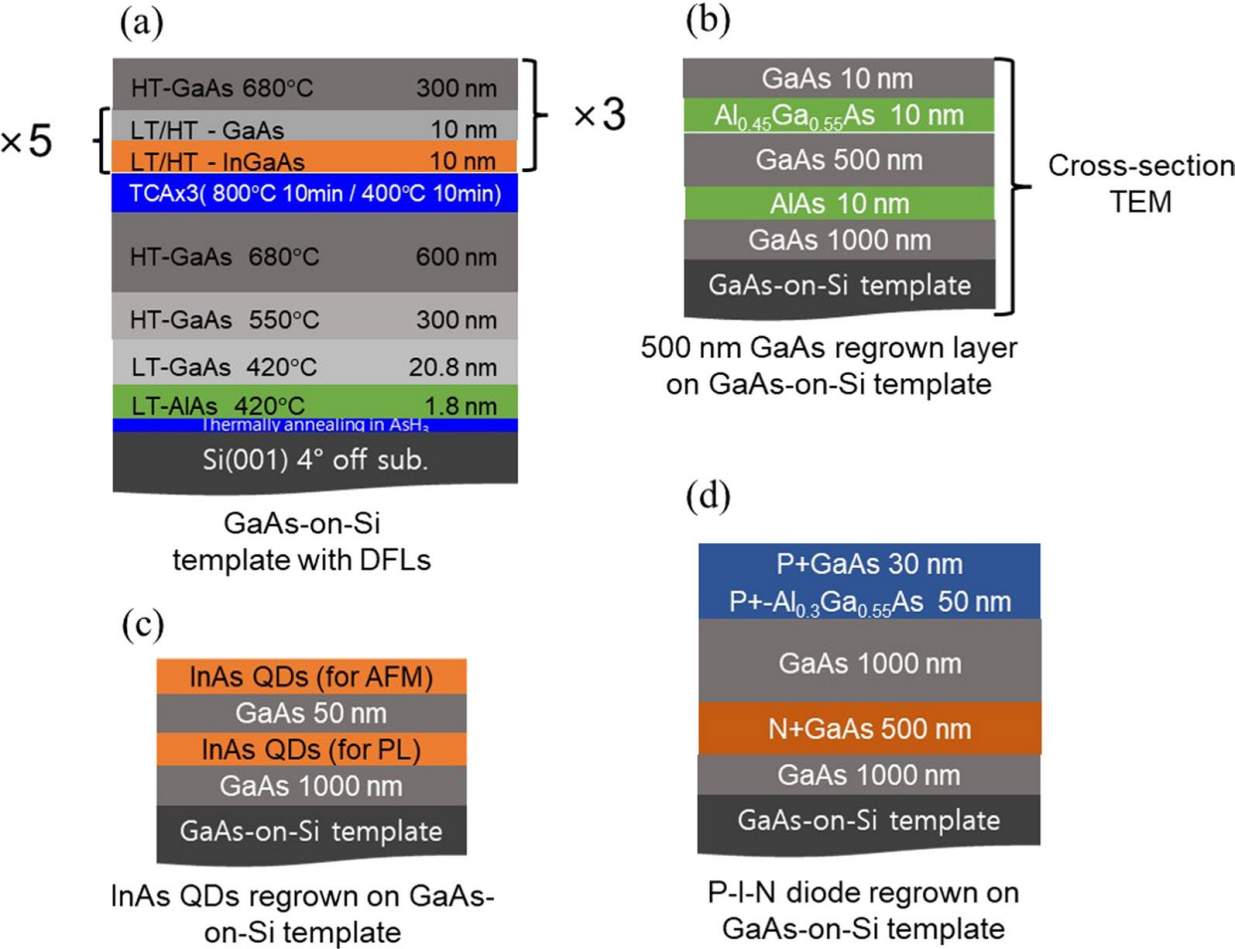
Schematic structures of GaAs-on-Si templates and regrown GaAs buffer and InAs QD layers. The TEM was observed using (**b**) structure and PL was evaluated using both the (**b**) and (**c**) structure, **a** Schematic structures of GaAs-on-Si templates with DFLs, **d** P-I-N diode regrown on GaAs-on-Si template

GaAs buffer layers were grown on 2-inch Si (100) substrates with a 4° miscut angle and all GaAs-on-Si templates were grown at the same time. Before starting the GaAs growth, the Si substrates were dipped in HF, then rinsed in deionized water and heated to 900 °C in H_2_ and AsH_3_ rich atmosphere. The 900 nm GaAs layers were then grown by the three-step growth method [20], with an AlAs initial seed layer [21, 22], followed by thermal cycling annealing (TCA). TCA was conducted between 800 and 350 °C. After TCA, the three sets of In_0.15_Ga_0.85_As/GaAs DFLs and 300 nm GaAs spacer layers were grown. The DFLs were grown at 550 °C low-temperature (LT)-DFL and 660 °C high-temperature (HT)-DFL and a control sample without DFLs was also grown to compare the defect level of GaAs-on-Si. The InGaAs/GaAs DFLs grown at LT and HT were calibrated by growing a superlattice at each temperature to keep the same composition and thickness. The defect level of the 2-μm-thick GaAs-on-Si templates was analyzed by ECCI. The TDD values were derived from ECCI measurement of four different wafer positions. After growth of the 2-μm-thick GaAs buffer, a 500 nm GaAs layer and InAs QDs were regrown on each sample with an additional 1 μm GaAs buffer to observe the optical characteristics of the regrown layer on GaAs on the Si template. The regrown 500 nm GaAs layer was sandwiched between an AlAs layer and Al_0.45_Ga_0.55_As layer and the QD layers were composed of a dot-in-a-well structure. A schematic of the sample structures is presented in Fig. 1b and c. The TD movement of the regrown 500 nm GaAs samples was observed by TEM and room-temperature (RT) PL was measured for the regrown 500 nm GaAs bulk layer and QD layer. The TEM was measured with a bright-field and a two-beam condition. For PL spectroscopy, a 532 nm diode-pumped solid-state laser and a cooled InGaAs detector were used. To analysis the electrical characteristics, the GaAs *p*–*i*–*n* diodes were regrown on the GaAs-on-Si templates as shown in figure (d) and fabricated through the standard fabrication process. The dark current of the diodes was measured in a black box using a semiconductor parameter analyzer (HP4156A). The bulk leakage current and the surface leakage current were extracted from the dark current measurements.

## Results and Discussion

**Fig. 2:**
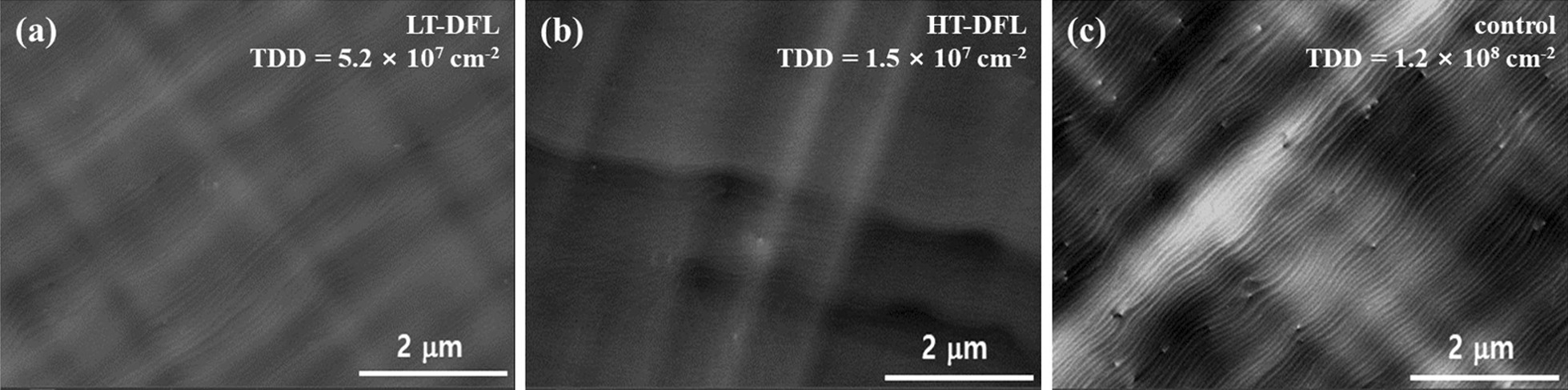
ECCI images of GaAs-on-Si templates with DFLs and without DFLs. **a**, **b** ECCI images of LT-DFL and HT-DFL, respectively. **c** ECCI image of GaAs-on-Si without DFLs as a control sample

Figure 2 shows the ECCI images of GaAs-on-Si templates with DFLs and without DFLs. The average TDD values of LT-DFL, HT-DFL and the control sample were 5.2 × 10^7^ cm^−2^, 1.5 × 10^7^ cm^−2^ and 1.2 × 10^8^ cm^−2^, respectively. As we assumed above, the DFLs grown at HT show the greatest reduction of the TDD level and clearly both DFLs show suppression of TD compared to the GaAs-on-Si without DFLs. Note that DFL samples have only three sets of DFL and the DFL only consists of five loops of superlattice layers. This structure has small numbers of sets and loops compared to the reported studies [12–15]. Therefore, the TDD values are slightly higher than the level of 10^6^ cm^−2^ where the GaAs buffer has five sets of DFLs and 10 loops of the superlattice layer. The TDD level can be further reduced, if the sets and loops are increased.

**Fig. 3:**
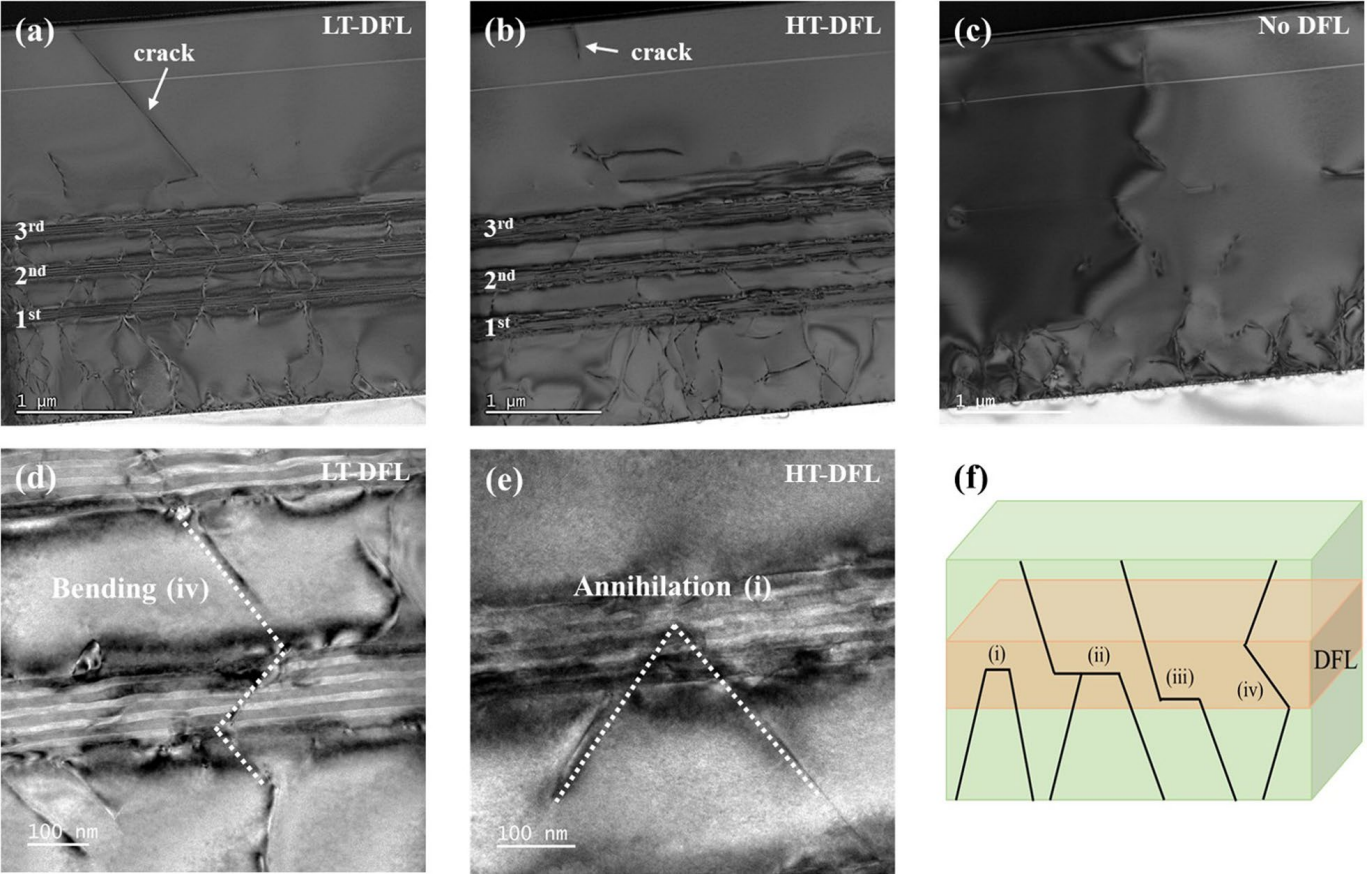
Cross-sectional TEM images and a schematic illustration of the dislocation motion. **a**, **d** LT-DFL and **b**, **e** HT-DFL, **c** depicts the TD propagation in the control sample which does not have DFLs, **f** is a schematic illustration of interaction and movement of TDs

Figure 3a–e presents cross-sectional TEM images of the TD propagation and Fig. 3f depicts a schematic illustration of interaction and movement of TDs. The interactions of TDs in the LT-DFL and HT-DFL are displayed in Fig. 3a, d and b, e, respectively. The control sample is shown in Fig. 3c. The propagation of TDs is clearly observed, reaching the 500 nm regrown GaAs layer without DFLs. As shown in Fig. 3a–b, the TDs are reduced whenever the TDs pass through each set of the DFLs, and the TDs in the HT-DFL are effectively annihilated compared to the TDs in LT-DFL. The dislocation interaction at the DFLs can be shown in four cases as illustrated in Fig. 3f. Case (i) is ‘annihilation’ where the two TDs combines into one MD and propagate toward the wafer edge or eliminate each other. Case (ii) is ‘fusion,’ where the two TDs combine into one MD and generate the one TD. Case (iii) is a ‘recycle + propagation’ process, where the direction of TD is converted to that of the MD and regenerates the TD, and case (iv) is ‘bending’ where the TD does not meet with the MD and passes through the DFLs. Cases (i) and (ii) can reduce the TDD. In the LT-DFL sample, the bending of TDs is more widely observed than the other processes, as shown in Fig. 3d. On the contrary, the annihilation of TDs in the HT-DFL is dominant, as depicted in Fig. 3e. These findings support that the high glide velocity of the TD caused by the high growth temperature of the DFLs induces active interaction with the MD and increases the possibility of TD annihilation.

**Fig. 4:**
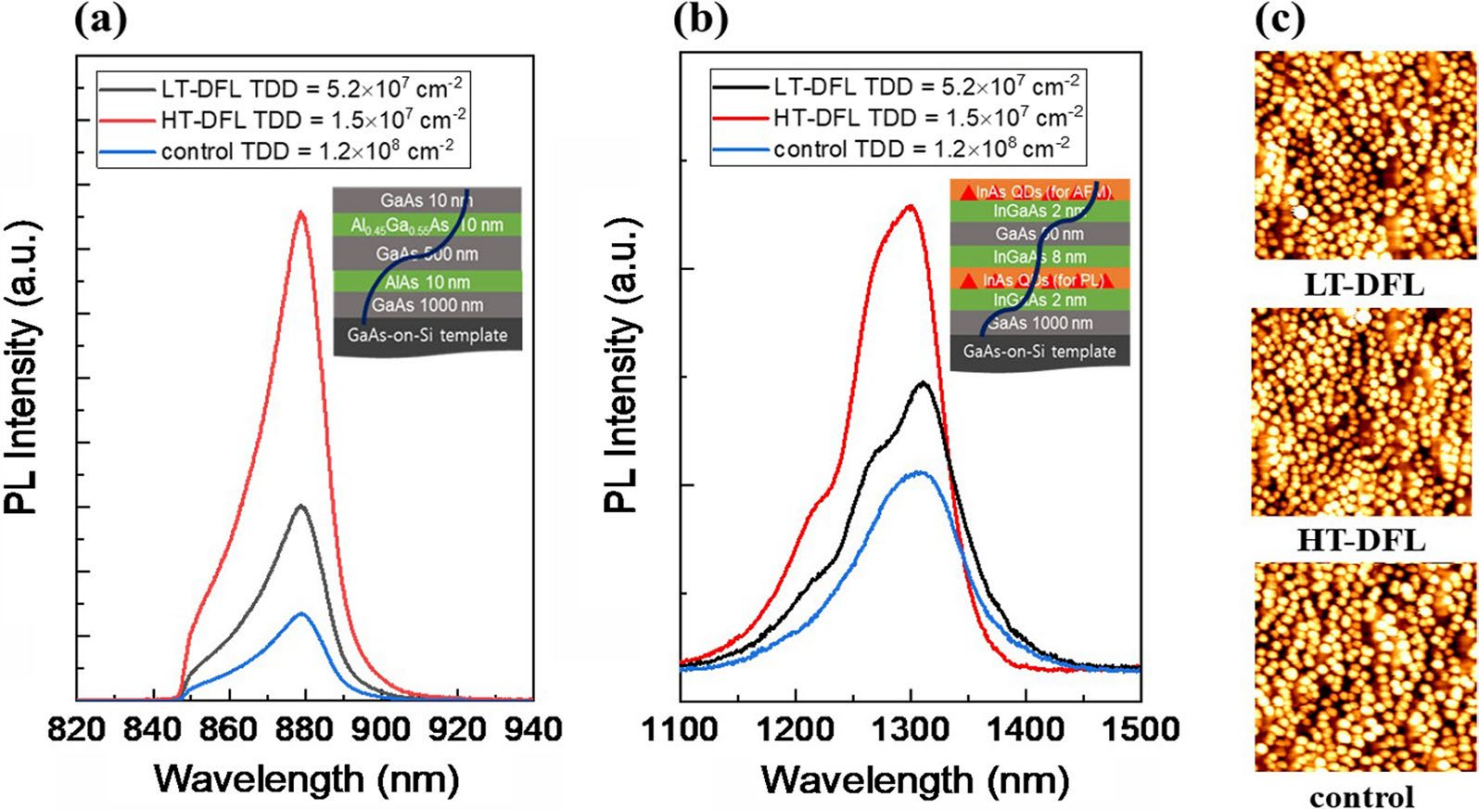
**a**, **b** PL results of a regrown 500 nm GaAs bulk layer and InAs QDs, respectively. The insets are illustrations of how TD affects the GaAs buffer and InAs QDs. **c** AFM images of regrown QDs on GaAs-on-Si templates

Figure 4a presents the PL results of a regrown 500 nm GaAs bulk layer on a GaAs-on-Si template. The integrated PL intensity of the HT-DFL shows the highest intensity, which is 250% and 560% higher than that of the LT-DFL and the control sample, respectively. The degree of the TDD level difference and the PL intensity difference shows good agreement, indicating that the PL of the GaAs buffer layer is significantly affected by the TDD level. This result is consistent with a previous report by Jung [19]. Figure 4b shows the PL results of regrown InAs QDs on the GaAs-on-Si template. Unlike the GaAs buffer results, the PL peak intensity does not show a tremendous difference between the samples, but only shows small differences. The PL peak intensity of the HT-DFL is156% and 216% higher than that of the LT-DFL and the control sample, respectively. The insets of Fig. 4a and b are illustrations of how the TD affects the GaAs buffer layer and InAs QDs. The TD can affect the entire GaAs bulk layer, resulting in the PL differences. On the other hand, each QD is isolated and a large number of ‘survived’ QDs, which are not affected by TD, can emit PL, resulting in a small difference in PL peak intensity. This indicates that the QDs are more resistant to the influence of TD than the GaAs bulk layers. Although the PL peak intensity did not show a large difference, the lifetime of InAs QDLD on Si shows a tremendous difference [18]. According to an earlier work by Jung [14], a 38-fold decrease of TDD shows an approximately fivefold increment of the laser lifetime. With respect to the slight differences in PL spectra among the QD samples, the reasons for the effects of dislocations or the inhomogeneous growth of QDs by MOVPE remain unclear, but we believe that this may be due to a combination of the factors noted above. Figure 4c shows AFM images of QDs grown on GaAs-on-Si templates and all QDs have a similar density level of 3.5 × 10^10^ cm^−2^. Since the PL intensity is mostly determined by the density of QDs, the highest PL peak intensity of HT-DFL is attributed to the reduced TDD and less non-radiative recombination occurring in the QD layer.

**Fig. 5:**
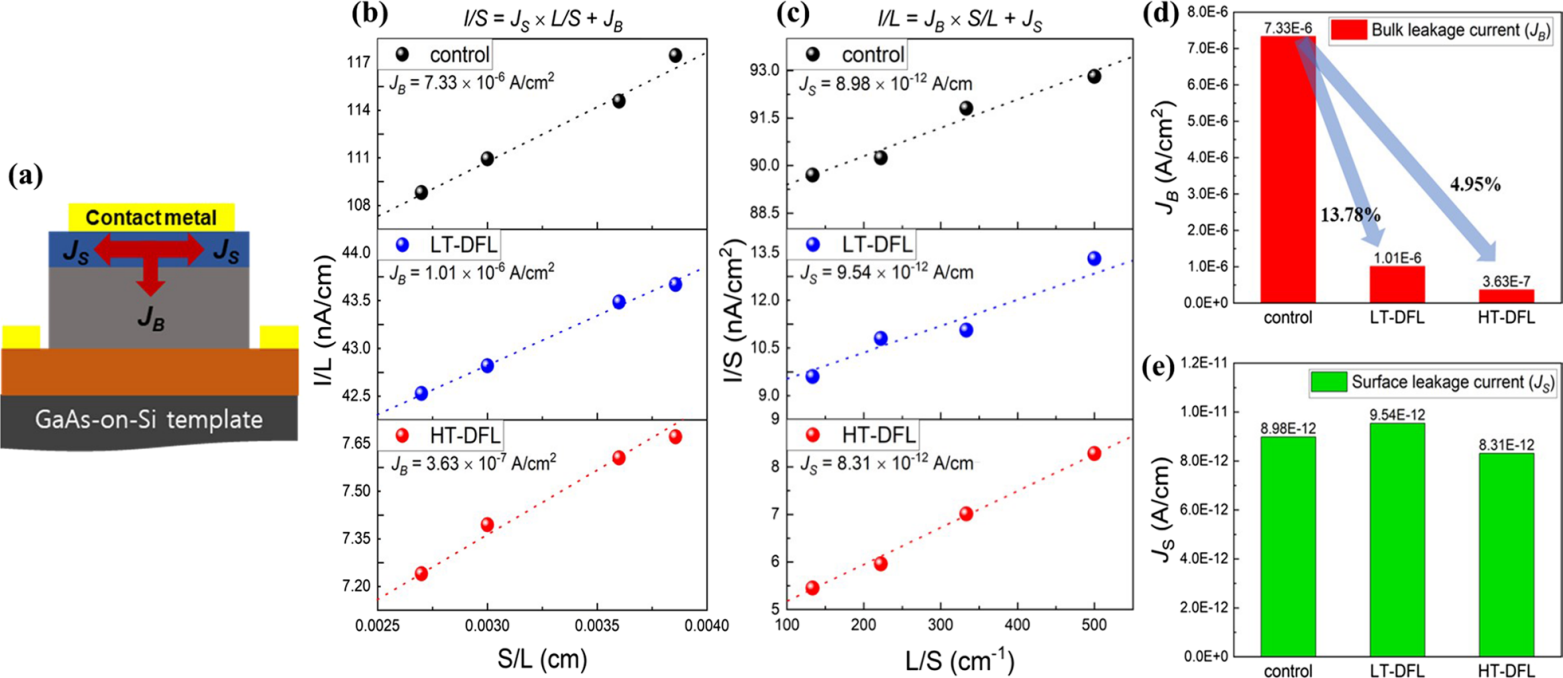
**a** Schematic of two possible dark current paths in a *p*–*i*–*n* GaAs diode on Si. **b** *S/L–I/L* relationship of GaAs *p*–*i*–*n* diode on Si at 1 V with various pattern sizes. **c** *L/S–I/S* relationship of GaAs *p*–*i*–*n* diode at 1 V with various pattern sizes. **d**, **e** Extracted values of *J*_B_ and *J*_S_ of the HT-DFL, LT-DFL and control sample, respectively

Figure 5a depicts the two dominant current paths in a diode, which are the bulk leakage current per junction area (*J*_B_) and the surface leakage current per peripheral length of junction (*J*_S_). The *J*_B_ is mainly determined by the crystal quality and this value becomes larger as the defect density is increased in a diode. The *J*_S_ usually shows huge differences by the device fabrication process such as a surface passivation. The total current can be expressed as *I* = *J*_B_ × *S* + *J*_S_ × *L*, where *I*, *S* and *L* are total current, junction area and peripheral length of the junction, respectively [23]. The *J*_B_ and *J*_S_ were extracted from the slope in the *S/L–I/L* and *L/S–I/S* relationship as shown in Fig. 5b and c. Both the *J*_B_ and *J*_S_ were extracted at applied bias of 1 V by the data fit with 4 data points. Extracted *J*_B_ of HT-DFL, LT-DFL and control sample is 3.625 × 10^–7^ A/cm^2^, 1.006 × 10^–6^ A/cm^2^ and 7.327 × 10^–6^ A/cm^2^, respectively, indicating that samples with DFL shows the decreased bulk leakage due to the annihilation process. The *J*_B_ of HT-DFL and LT-DFL shows the 4.95% and 13.78% of decrease, respectively, compared to that of the control sample as shown in Fig. 5d. The GaAs *p*–*i*–*n* diode on HT-DFL has the lowest bulk leakage current among the samples, which shows a good agreement with the PL results. On the other hands, extracted *J*_S_ of HT-DFL, LT-DFL and control sample is 8.31 × 10^–12^ A/cm, 9.544 × 10^–12^ A/cm and 8.984 × 10^–12^ A/cm, respectively, showing a same level as depicted in Fig. 5e. The same level of *J*_S_ indicates that all diode was successfully fabricated with a same device fabrication condition. The noticeable difference of *J*_B_, which is dominantly affected by TDD from GaAs-on-Si templates, implying that the high-temperature growth of DFL can make a low electrical loss of GaAs *p*–*i*–*n* diodes on Si. From these results, the high-temperature growth of DFLs may be useful for good performance of QDLDs on a Si substrate grown by MOVPE.

## Conclusions

In conclusion, we have investigated the GaAs-on-Si templates with DFLs grown by MOVPE with varying growth temperature. To produce high glide velocity of TD, the DFLs were grown at a high temperature of 660 °C and compared with those grown at a low temperature of 550 °C. The ECCI shows a fivefold lower TDD value of 1.5 × 10^7^ cm^−2^ of the HT-DFL than that of the LT-DFL. The annihilation process was mainly observed, caused by the high degree of motion of TDs. On the other hand, the bending process was mostly observed, resulting in a higher TDD value of 5.2 × 10^7^ cm^−2^. The GaAs buffer layer and InAs QDs were regrown on GaAs-on-Si templates and both the PL of the GaAs buffer and QDs on the HT-DFL show the highest intensity, indicating that the reduced TDD generated less non-radiative recombination. The GaAs *p*–*i*–*n* diodes were also regrown on GaAs-on-Si templates and the *J*_B_ was extracted from the dark current measurement. The *J*_B_ of HT-DFL shows the 4.95% of decrease compared to that of the control sample, which is the lowest value among the samples. This implies that high-temperature growth of DFL can make a good performance GaAs device on Si. These results provide an avenue for further improvement of growth of GaAs-on-Si templates.

## Data Availability

The datasets used and/or analyzed during the current study are available from the corresponding author on reasonable request.
